# Wharton's Jelly Mesenchymal Stromal Cell‐Derived Extracellular Vesicles Attenuate Intervertebral Disc Degeneration Under Inflammatory Stress in an In Vitro 3D Culture System

**DOI:** 10.1002/jsp2.70106

**Published:** 2025-08-20

**Authors:** Veronica Tilotta, Gianluca Vadalà, Giuseppina Di Giacomo, Luca Ambrosio, Claudia Cicione, Fabrizio Russo, Adas Darinskas, Rocco Papalia, Vincenzo Denaro

**Affiliations:** ^1^ Laboratory for Regenerative Orthopaedics, Operative Research Unit of Orthopaedic and Trauma Surgery Fondazione Policlinico Universitario Campus Bio‐Medico Rome Italy; ^2^ Research Unit of Orthopaedic and Trauma Surgery, Departmental Faculty of Medicine and Surgery Università Campus Bio‐Medico di Roma Rome Italy; ^3^ Laboratory of Immunology National Cancer Institute Vilnius Lithuania; ^4^ JSC Innovita Research Tissue Bank Vilnius Lithuania

**Keywords:** exosome, extracellular vesicles, intervertebral disc, intervertebral disc degeneration, intervertebral disc regeneration, low back pain, mesenchymal stromal cells

## Abstract

**Background:**

Extracellular vesicles (EVs) derived from mesenchymal stromal cells (MSCs) are emerging as a promising cell‐free strategy for intervertebral disc degeneration (IDD) treatment. This study aimed to evaluate the anabolic effect of Wharton's Jelly MSC (WJ‐MSC)‐derived EVs on degenerative human nucleus pulposus cells (hNPCs) under in vitro inflammation using a 3D culture model.

**Methods:**

Following isolation, hNPCs (*n* = 10) were encapsulated in alginate beads and treated with 10, 50, and 100 μg/mL of WJ‐MSC‐EVs after preincubation with 10 ng/mL interleukin (IL)‐1β. Cell proliferation, viability, nitrite production, and glycosaminoglycan (GAG) content were assessed. Histological analyses evaluated extracellular matrix (ECM) production. Phenotypic (SOX9, KRT19), catabolic (MMP1, MMP13, ADAMTS5, IL6, NOS2), and anabolic (ACAN) ECM markers were analyzed by RT‐qPCR.

**Results:**

WJ‐MSC‐EVs significantly promoted hNPC proliferation at all concentrations, with 10 μg/mL effectively counteracting IL‐1β catabolic effects. Live/dead staining showed reduced cell death in EV‐treated hNPCs compared to the IL‐1β‐only group. Nitrite production decreased after 7 days with 10 μg/mL WJ‐EVs, supported by reduced NOS2 expression. GAG content increased dose‐dependently, as confirmed by Alcian blue staining. WJ‐EVs positively modulated anabolic (ACAN, KRT19, SOX9), catabolic (ADAMTS5, MMP1, MMP13), and inflammatory (IL6) gene expression levels.

**Conclusion:**

WJ‐MSC‐derived EVs demonstrate potential as a cell‐free therapeutic approach for IDD by enhancing hNPC growth, mitigating ECM degradation, and reducing oxidative stress‐related IDD progression. These findings warrant further investigation into the use of WJ‐EVs for IDD treatment.

This study explores the therapeutic potential of extracellular vesicles (EVs) derived from Wharton's Jelly mesenchymal stem cells in an in vitro 3D model of intervertebral disc degeneration under inflammatory stress. The treatment with WJ‐MSC‐EVs enhanced nucleus pulposus cell proliferation, viability, and extracellular matrix synthesis while reducing oxidative stress and catabolic gene expression. These results support the promise of WJ‐MSC‐EVs as a novel, cell‐free strategy for disc regeneration in inflammatory conditions.

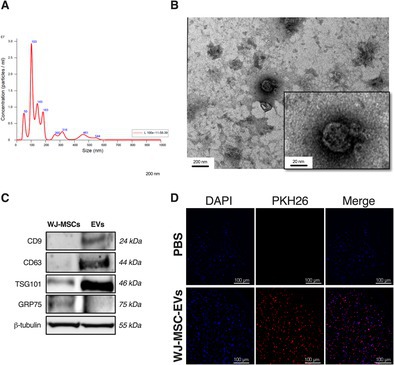

## 1 Introduction

Low back pain (LBP) is the leading cause of disability worldwide [1, 2], with intervertebral disc degeneration (IDD) as its primary trigger [3]. The prevalence of IDD is influenced by several factors including aging, mechanical load imbalances, obesity, genetic predisposition, smoking, trauma, and inflammation [4, 5]. During this process, the inner, gel‐like, and shock‐absorbing nucleus pulposus (NP) undergoes progressive depletion of resident cells, extracellular matrix (ECM) breakdown, and dehydration. As the intervertebral disc (IVD) height gradually decreases, biomechanical alterations affect adjacent tissues, leading to potential complications like disc herniation, spinal stenosis, or instability, which may result in significant pain and disability [6].

Current treatments for IDD primarily aim to relieve pain and improve quality of life through physical therapy, analgesics, epidural steroid injections, or surgery. However, these approaches often provide only limited or temporary relief. In recent decades, the possibility of directly counteracting the degenerative process by introducing functional cells into damaged IVDs has yielded promising results [7]. Mesenchymal stromal cells (MSCs) are particularly promising for IDD therapy due to their safety profile, ease of harvesting (e.g., bone marrow, adipose tissue, umbilical cord, Wharton's Jelly, etc.), and regenerative potential [8]. Indeed, MSCs are widely applied in regenerative medicine for their ability to repair tissue damage and their immunomodulatory properties [9]. They produce a “cocktail” of paracrine factors known as the secretome, which includes extracellular vesicles (EVs), cytokines, and growth factors [10]. The MSC secretome and EVs are rich in molecules with pro‐regenerative effects, promoting immune modulation, inhibiting cell death and fibrosis, enhancing tissue remodeling, and recruiting additional cells [11]. EVs, especially exosomes and microvesicles, can “reprogram” target cells by delivering proteins, lipids, and nucleic acids. Recent studies have demonstrated that EVs promote cell proliferation, inhibit nucleus pulposus cell (NPC) apoptosis, and support ECM synthesis, potentially delaying IDD progression [12].

Wharton's Jelly‐derived MSCs (WJ‐MSCs) are collected from the umbilical cord's gelatinous tissue, considered medical waste after childbirth, and can be obtained via a noninvasive and painless process. WJ‐MSCs offer unique advantages, including ethical acceptability, differentiation potential, high proliferative capacity, immunomodulatory effects, and a low risk of tumorigenesis or graft‐versus‐host reactions, making them highly suitable for regenerative applications [13]. Unlike adult MSCs, which may face clinical limitations due to invasive collection and limited donors, WJ‐MSCs provide a versatile therapeutic option. Given their properties, it is plausible to speculate that WJ‐MSCs produce EVs with a rich regenerative cargo. Indeed, we have previously shown that WJ‐MSC‐EVs were able to enhance degenerative human NPC (hNPC) proliferation, metabolism, and GAG content in vitro [14]. However, the effects of these EVs under pro‐inflammatory, IDD‐like conditions remain unexplored.

This study aimed to explore the effects of WJ‐MSC‐derived EVs on hNPC proliferation, viability, and ECM production under inflammatory conditions, using an in vitro 3D alginate‐bead culture model to mimic the adverse microenvironment of the degenerative IVD.

## 2 Methods

### 2.1 Isolation and Culture of hNPCs

The study was conducted in accordance with the Declaration of Helsinki, and the protocol was approved by the Ethics Committees of Università Campus Bio‐Medico di Roma (Rome, Italy) and National Cancer Institute (Vilnius, Lithuania) under approval numbers n. 09/15 PAR ComEt CBM and n. 2021/01‐1301‐779, respectively. Nucleus pulposus specimens were obtained from 10 patients (mean age: 46.0 ± 16.3 years, no comorbidities; Table 1) undergoing discectomy for lumbar disc herniation at Fondazione Policlinico Universitario Campus Bio‐Medico (Rome, Italy) [14]. Patients with endplate or Modic changes at the index level and/or a history of prior spine surgery were excluded. All patients provided informed consent for the collection and use of surgical tissues for research purposes. Specimen processing followed standard procedures detailed in our previous work [15]. Briefly, after enzymatic digestion with collagenase, the cell suspension was filtered through a 70‐μm filter, centrifuged at 300 g for 5 min, and the isolated cells were cultured in Dulbecco's Modification of Eagle's Medium High Glucose (DMEM; Corning, Corning, NY, USA) supplemented with 10% fetal bovine serum (FBS; Gibco, Waltham, MA, USA), 1% penicillin/streptomycin (P/S), 1% glutamine (Sigma, St. Louis, MO, USA), and 25 μg/mL ascorbic acid under standard culture conditions (21% O_2_, 5% CO_2_, 37°C). The culture medium was refreshed every 3 days, and cells were grown to 80%–90% confluency before further use.

**TABLE 1:** Summary of characteristics of the donor IVD samples.

Patient ID	Age (years)	Sex	Level	Pfirrmann grade
1	58	M	L5‐S1	IV
2	39	F	L5‐S1	IV
3	47	M	L3‐L4	III
4	55	F	L4‐L5	III
5	75	F	L4‐L5	IV
6	37	M	L4‐L5	III
7	53	F	L5‐S1	III
8	67	F	L4‐L5	IV
9	63	F	L4‐L5	IV
10	54	M	L4‐L5	III

*Note:* Age, sex, operated levels, and Pfirrmann grading are listed.

Abbreviation: IVD, intervertebral disc.

### 2.2 Isolation and Characterization of WJ‐MSCs

Human WJ‐MSCs were obtained from the National Cancer Institute (Lithuania) and characterized as previously described [14]. Briefly, healthy donors (mothers aged 18–44 years) signed an informed consent to agree with the use of their umbilical cord tissue for research purposes. Several batches of WJ‐MSCs, at passages 4–6 (P4–P6), were obtained from a single donor. Cells were seeded at a density of 4 × 10^3^ cells/cm^2^ in DMEM Low Glucose with 10% FBS, 2 mM L‐Alanyl‐L‐Glutamine, and 40 μg/mL gentamicin, and cultured in uncoated five‐layer cell culture Multi‐Flasks (Corning) until reaching 70%–80% confluence. Cells were then washed three times with 50 mL PBS without Ca^2+^ or Mg^2+^ and re‐cultured in DMEM High Glucose (without phenol red) supplemented with 2 mM L‐Alanyl‐L‐Glutamine, 2% platelet‐rich plasma (PRP) lysate (PLTGold Human Platelet Lysate, Sartorius, Göttingen, Germany), 2 IU/mL heparin, and 40 μg/mL gentamicin under standard conditions (21% O_2_, 5% CO_2_, 37°C) for 1–2 days. To confirm cell identity, flow cytometry analysis assessed the positivity for CD73, CD90, and CD105, and negativity for SSEA3, with a minimum of 25000 cell events recorded using a CytoFLEX cytometer (Beckman Coulter, Brea, CA, USA). The multilineage differentiation potential of WJ‐MSCs was evaluated through culture in osteogenic, adipogenic, and chondrogenic media [10]. Osteogenic medium contained DMEM Low Glucose with 10% FBS, 0.1 μM dexamethasone, 0.2 mM ascorbic acid 2‐phosphate, and 10 mM glycerol 2‐phosphate. For adipogenic differentiation, DMEM Low Glucose was supplemented with 10% FBS, 1 μM dexamethasone, 0.5 mM 3‐isobutyl‐1‐methylxanthine (IBMX), 10 μg/mL insulin, and 100 μM indomethacin. Chondrogenic conditions were achieved by trypsinizing WJ‐MSCs, centrifuging at 300 g for 5 min to form cell pellets, and culturing in Chondrogenic Differentiation Medium (Lonza, Switzerland). After 21 days, Alizarin Red and Oil Red O stains were applied to WJ‐MSCs in monolayer to assess osteogenic and adipogenic differentiation, respectively. Cell pellets were fixed in 10% neutral buffered formalin, paraffin‐embedded, and stained with Alcian Blue to assess chondrogenic differentiation. Microscopic evaluation was conducted using a NanoZoomer 2.0RS (Hamamatsu Photonics, Hamamatsu, Japan) at 10× and 20× magnifications. Six random fields per histological section were digitally captured, and staining intensities (Alizarin Red and Alcian Blue for ECM or Oil Red O for lipid vacuoles) were independently analyzed by three blinded evaluators to determine WJ‐MSC multidifferentiation potential.

### 2.3 Isolation and Characterization of WJ‐MSC‐Derived EVs

WJ‐MSCs were isolated and characterized as previously described [14]. Once reaching 70%–80% confluency, WJ‐MSCs were washed with PBS and cultured in serum‐free medium for 24 h at 37°C in a 5% humidified CO_2_ environment. The conditioned medium was collected and passed through a 0.22 μm filter to eliminate cell debris. EVs were then isolated from the filtered medium using tangential flow filtration with a VivaFlow 200 filter and a 100 kDa molecular weight cutoff, concentrating the solution 10‐fold. The buffer was exchanged for PBS, and the final product was sterile‐filtered using a 220 nm filter (Sigma).

To characterize the EVs, their concentration and size distribution were measured by nanoparticle tracking analysis (NTA) using the NANOSIGHT NS300 system (Malvern Panalytical, Malvern, UK) following the manufacturer's protocol. EV morphology was assessed by transmission electron microscopy (TEM). For TEM preparation, EVs suspended in PBS were loaded onto formvar carbon‐coated copper grids and allowed to adsorb for 10 min at room temperature. The grids were then negatively stained with 1% phosphotungstic acid for 5 min, air‐dried, and observed under TEM to visualize EV structure. EV protein and DNA concentrations were quantified using the Bradford assay and the Qubit dsDNA Quantification assay (Thermo Fisher), respectively.

### 2.4 hNPCs Encapsulation in Alginate Beads and Treatment With EVs

hNPCs were grown in a three‐dimensional system as previously described [15]. Cultures were maintained in DMEM with 10% FBS, 1% P/S, and 25 μg/mL ascorbic acid in a 5% CO_2_ and 95% air incubator. After 2 weeks, the cultures were divided into four treatment groups: (1) DMEM with EV‐free serum as a control; (2) WJ‐MSC‐EVs concentrations at 10, 50, or 100 μg/mL; (3) 10 ng/mL interleukin (IL)‐1β (Proteintech Group Inc., Rosemont, IL, USA) to mimic the inflammatory IDD microenvironment; (4) 10 ng/mL IL‐1β for 24 h (day −1), followed by co‐incubation with WJ‐MSC‐EVs at 10, 50, or 100 μg/mL. To prepare EV‐free serum, FBS was ultracentrifuged at 110,000 g for 17 h, and the supernatant was filtered through a 0.22‐μm filter, as described previously [14]. The media were replenished every 2 days.

### 2.5 Cell Count and Viability

hNPCs in alginate beads (*n* = 4) were dissolved by incubation in 55 mmol/L sodium citrate, 30 mmol/L EDTA, 0.15 M NaCl, and pH 6.8 for 10 min at 4°C. Cell proliferation and viability were analyzed using a CytoFLEX cytometer with the CytExpert Software (v.2.1, Beckman Coulter). Cell count was assessed at 1, 4, 10, and 14 days expressed as event/μL. To evaluate cell death, hNPCs were stained with the Fixable Viability Dye conjugated with eFluor780 fluorochrome (Affimetrix eBioscience, Thermo Fisher Scientific) after 24 h of treatment as previously described [14]. Likewise, cell viability was assessed with the LIVE/DEAD assay following the manufacturer's instructions. Briefly, hNPCs in 3D (*n* = 4) were incubated for 45 min with ethidium homodimer‐2 and calcein acetoxymethylester at room temperature and washed three times with PBS and Hoechst 33258 (Life Technologies, Thermo Fisher Scientific) to stain the nuclei. After incubation, green, red, and blue fluorescence was detected using a confocal laser scanning microscope (Zeiss LSM700, Carl Zeiss Germany). Z‐stacks were acquired across the whole bead volume, with optical slices being combined through maximum intensity projection. Cell viability was then quantified with ImageJ (v. 1.54).

### 2.6 hNPC Nitrite Concentration

The Griess reaction (Invitrogen, Carlsbad, CA, USA) was used to quantify nitrite levels in the supernatant, as an indicator of nitric oxide (NO) production. In this reaction, nitrites in the medium react with sulfanilic acid under acidic conditions, forming a purple azo compound that subsequently couples with naphthyl‐ethylenediamine dihydrochloride dissolved in water. After 1 week of treatment, supernatant samples from both treated hNPCs and controls (*n* = 5) were incubated with 20 μL of Griess reagent for 30 min in the dark, following the manufacturer's protocol. Absorbance was then measured at 546 nm using Tecan Infinite M200 PRO. Nitrite concentrations were calculated against a standard curve, with results normalized to the control group.

### 2.7 1,9‐Dimethylmethylene Blue Assay

GAG content was evaluated as previously described [14]. After 7 days of treatment, alginate beads were dissolved, and the entire solution including hNPCs (*n* = 8) and the surrounding ECM were digested overnight at 65°C in 100 μL of papain solution (0.25 mg/mL in 50 mM phosphate buffer, pH 1.5) containing 5 mM cysteine hydrochloride and 5 mM EDTA. GAG levels were quantified by reaction with dimethylmethylene blue (DMMB; Polysciences, Warrington, PA, USA), using chondroitin sulfate (Sigma) as the standard. Absorbance was measured at 530 nm with a Tecan Infinite M200 PRO. GAG content was normalized to DNA levels, allowing for comparison of percentage changes between the control (baseline) and treatment groups. DNA content was determined using a PicoGreen assay (Invitrogen), with quantification based on a DNA standard curve. Fluorescence was measured at excitation and emission wavelengths of 488 and 520 nm, respectively, using a microplate reader (Tecan Infinite M200 PRO).

### 2.8 Histological Evaluation

After 7 days of treatment, alginate beads were fixed in 10% (v/v) phosphate‐buffered formalin (Sigma), paraffin‐embedded, and sectioned at 5 μm thickness following standard protocols. Consecutive sections were dewaxed, rehydrated, and stained with hematoxylin–eosin to examine cell morphology and Alcian Blue to assess proteoglycan content. Microscopic analysis was conducted at 10× and 20× magnification using a NanoZoomer 2.0RS (Hamamatsu Photonics).

### 2.9 RNA Extraction and Gene Expression Analysis

After 7 days of treatment, total RNA was isolated from pellets following alginate bead digestion using TRIzol reagent (Invitrogen). cDNA was synthesized using the High‐Capacity cDNA Reverse Transcription Kit (Applied Biosystems, Foster City, CA, USA), following the manufacturer's guidelines. mRNA levels of aggrecan (ACAN; Hs0153936), matrix metalloproteinase (*MMP*)*‐1* (Hs00899658), MMP‐13 (Hs00233992), a disintegrin and metalloproteinase with thrombospondin motifs (*ADAMTS*)*‐5* (Hs00199841) SRY‐Box Transcription Factor (*SOX*)*‐9* (Hs01001343), IL‐6 (Hs00174131), nitric oxide synthase (*NOS*)*‐2* (Hs01075529) and glyceraldehyde‐3‐phosphate dehydrogenase (*GAPDH*) (Hs03929097) were quantified using qRT‐PCR with TaqMan Gene Expression Assays and TaqMan Universal Master Mix II on a 7900HT FAST Real‐Time PCR System. For keratin (*KRT*)*‐19* (Forward: AAC GGC GAG CTA GAGGT GA; Reverse: GGA TGG TCG TGT AGT AGT GGC) and GAPDH (Forward: GAA GGT GAA GGT CGG AGT; Reverse: GAA GAT GGT GAT GGG ATT TC) expression analysis was performed using SYBR Green Master Mix (Applied Biosystems). GAPDH served as the reference gene to normalize target gene expression. The expression level of each gene was normalized to GAPDH expression and calculated as 2^−ΔΔCt^. Results for the experimental group were expressed relative to the control group, which was set as the baseline.

### 2.10 Western Blot

Total proteins were isolated from WJ‐MSC and WJ‐MSC‐EVs using the radioimmunoprecipitation assay buffer (RIPA buffer; Sigma) for 30 min on ice, cleared by centrifugation for 30 min at 12,000g at 4°C, and quantified using the detergent compatible (DC) protein assay kit (Bio‐Rad, Hercules, CA, USA). Samples were loaded on 4%–12% SDS‐PAGE gels, transferred onto nitrocellulose membranes through the Trans‐Blot Turbo Transfer System (Bio‐Rad) and incubated in a blocking buffer (TBST 1× with 5% nonfat dry milk) for 1 h. Membranes were incubated with a primary antibody overnight, shaking at 4°C in TBST 1× with 1% nonfat dry milk. Anti‐CD9 (mouse, 1:1000, Invitrogen), anti‐CD63 (mouse, 1:1000, Invitrogen), anti‐TSG101 (mouse, 1:1000, Invitrogen), anti‐GRP75 (rabbit, 1:2000, Proteintech, Thermo Fisher Scientific), and anti‐tubulin (1:5000, Abcam, Cambridge, UK) primary antibodies were used. Anti‐mouse and anti‐rabbit HRP‐conjugated antibodies (1:10000, Abcam) were utilized, and chemiluminescence signals were measured using ChemiDoc (Bio‐Rad) and Quantity One software (Bio‐Rad) to quantify the signal intensity of protein bands.

### 2.11 Statistical Analysis

Quantitative data are presented as mean ± standard deviation (SD). The normality of data distribution was assessed using the Shapiro–Wilk test. Statistical comparisons were conducted via one‐way or two‐way analysis of variance (ANOVA), applying Dunnett's or Tukey's post hoc tests as appropriate for multiple comparisons, with the IL‐1β group set as a reference. When multiple time points were considered, data were analyzed using unpaired *t* tests within groups. Details regarding the tests utilized for each single analysis are listed in the respective figure caption. A *p* value of less than 0.05 was considered statistically significant. All analyses were performed using Prism 10 software (GraphPad, San Diego, CA, USA). Each experiment was conducted in triplicate, with representative results shown.

## 3 Results

### 3.1 Characterization of WJ‐MSC‐EVs and WJ‐MSCs

**FIGURE 1:**
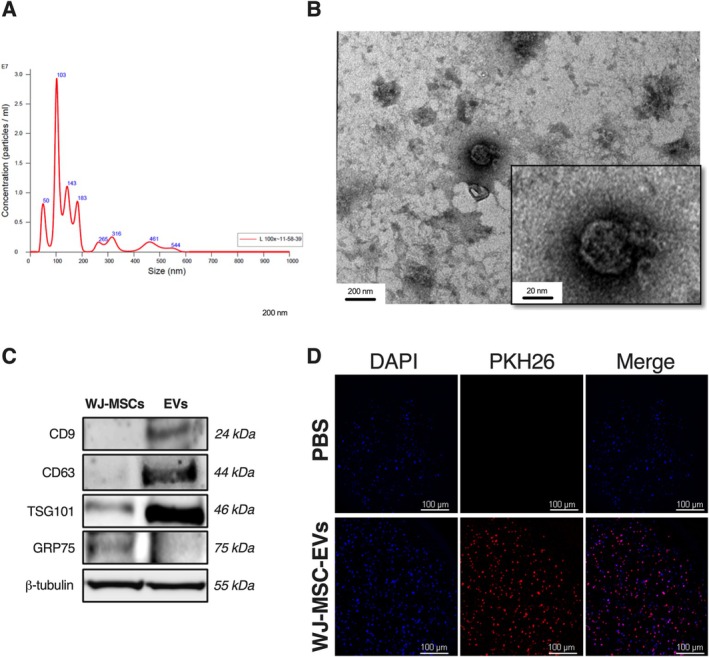
Characterization of WJ‐MSC‐EVs. (A) Particle size distribution of WJ‐MSC‐EVs measured with NTA. (B) TEM showed the typical EV morphology in WJ‐MSC‐EVs (left, scale bar: 200 nm; right box, scale bar: 20 nm). (C) WB analysis of EV protein markers CD63, CD9, and TSG101. (D) Representative images of hNPCs incubated with PBS or PKH26‐labeled WJ‐MSC‐EVs (red). hNPC nuclei were stained with DAPI (blue). Magnification: 20× scale bar: 100 μm. Abbreviations: DAPI, 4′,6‐diamidino‐2‐phenylindole, EVs, extracellular vesicles, hNPCs, human nucleus pulposus cells, NTA, nanoparticle trafficking analysis, PBS, phosphate‐buffered saline, TEM, transmission electron microscopy, WB, Western blot, WJ‐MSCs, Wharton's Jelly mesenchymal stromal cells. Reproduced with permission from Tilotta et al. [14].

**FIGURE 2:**
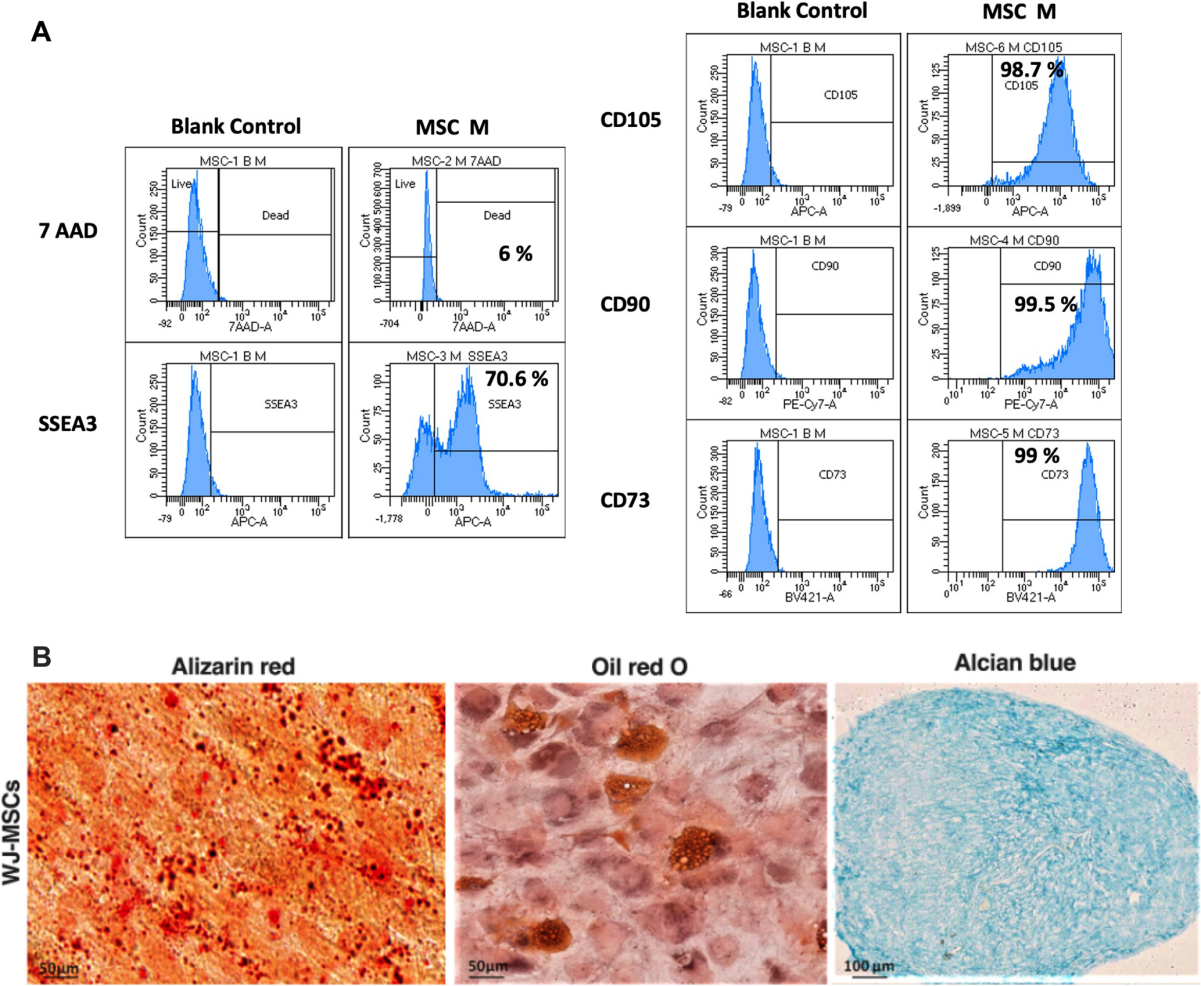
Characterization of WJ‐MSCs. (A) Surface markers (CD90, CD105, CD73, and SSEA3) detected through flow cytometric analysis. (B) Multilineage differentiation of WJ‐MSCs towards the osteogenic, adipogenic, and chondrogenic lineages was confirmed by Alizarin Red, Oil red O, and Alcian Blue staining (left and center, scale bars: 50 μm; right, scale bar: 100 μm). Abbreviation: WJ‐MSCs, Wharton's Jelly mesenchymal stromal cells. Reproduced with permission from Tilotta et al. [14].

The WJ‐MSC‐EVs and WJ‐MSCs utilized in this study have been characterized as previously reported [14]. The mean EV concentration was 1.60 × 10^9^ particles/mL, with an average diameter of 172.5 nm, an average protein concentration of 0.8 mg/mL, and an average DNA concentration of 57 ng/mL (Figure 1A). TEM confirmed the typical EV morphology (Figure 1B), whereas WB analysis showed a higher expression of EV surface markers TSG101, CD9, and CD63 in WJ‐MSC‐EVs compared to WJ‐MSC lysates (Figure 1C). PKH‐26‐labelled WJ‐MSC‐EVs were observed to localize in the perinuclear region of hNPCs, indicating effective internalization (Figure 1D). Flow cytometry analysis of WJ‐MSCs revealed positive expression for CD90 (97.2%), CD73 (92.6%), and CD105 (93.4%), with minimal SSEA3 expression (4.8%), confirming their MSC phenotype (Figure 2A). Differentiation potential was demonstrated by calcium deposition (Alizarin red staining), lipid droplet formation (Oil Red O staining), and GAG production (Alcian blue staining), indicating osteogenic, adipogenic, and chondrogenic lineage commitment, respectively (Figure 2B).

### 3.2 WJ‐MSC‐EVs Promoted Cell Proliferation and Viability

**FIGURE 3:**
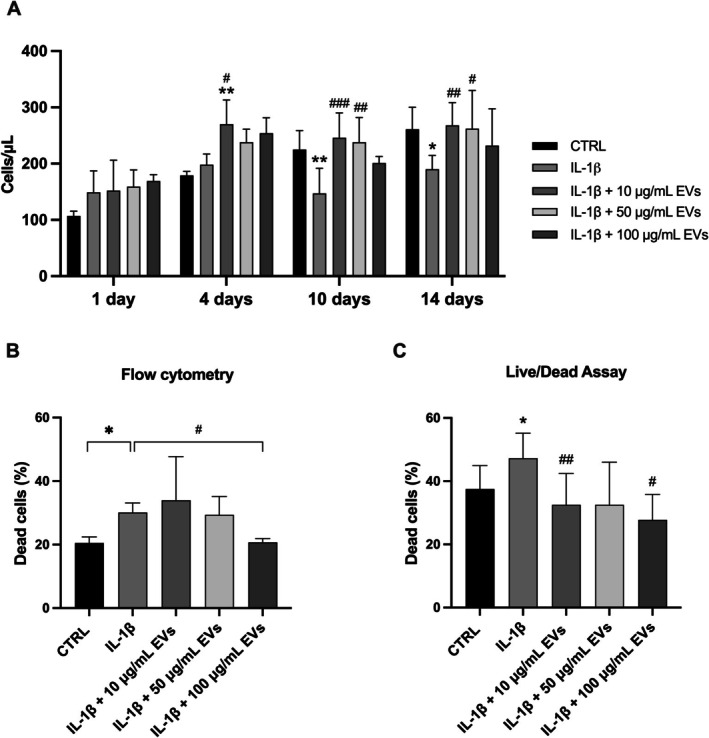
WJ‐MSC‐derived EVs promoted hNPC proliferation and viability. (A) Cell content significantly increased after treatment with 10 μg/mL WJ‐MSC‐EVs at day 4, 10, and 14, as compared with the IL‐1β control group (*n* = 4). Data were analyzed through two‐way ANOVA with Dunnett's multiple comparisons test, where each group was compared with IL‐1β. Within each timepoint, values were analyzed using unpaired t tests with a false discovery rate approach. (B) After 24 h, the WJ‐MSC‐EV treatment significantly reduced IL‐1β‐pre‐treated hNPC death at 100 μg/mL at flow cytometry (*n* = 4). Data were analyzed through two‐way ANOVA with Dunnett's multiple comparisons test, where each group was compared with IL‐1β. (C) Live/Dead staining showed reduced cell death in hNPCs treated with 10 and 100 μg/mL WJ‐MSC‐EVs (*n* = 4). Magnification: 20×, scale bar: 100 μm.*p < 0.05, **p < 0.01, compared to the control group. ^#^ *p* < 0.05, ^##^ *p* < 0.01, ^###^ *p* < 0.001 compared to the IL‐1β group. Abbreviations: AM, acetoxymethylester; hNPCs, human nucleus pulposus cells; WJ‐MSC‐EVs, Wharton's Jelly mesenchymal stromal cell‐derived extracellular vesicles.

Flow cytometry was performed to evaluate cell viability and proliferation following treatment with increasing doses of WJ‐MSC‐EVs (Figure 3A). On Day 4, pretreatment with 10 ng/mL IL‐1β for 24 h followed by the addition of 10 μg/mL WJ‐MSC‐EVs resulted in a significant increase in cell count (270.0 ± 21.9 cells/μL, *p* < 0.01) compared to the control group. In contrast, IL‐1β treatment alone reduced hNPCs proliferation at Days 10 and 14 (225.0 ± 28.1 cells/μL and 261.0 ± 23.0 cells/μL, respectively, *p* < 0.01). After 4, 10, and 14 days in 3D culture, the mean cell count of IL‐1β‐pre‐treated hNPCs exposed to 10 μg/mL WJ‐MSC‐EVs remained significantly higher (270.2 ± 23.6 cells/μL; 246.0 ± 31.4 cells/μL and 268.0 ± 23.60 cells/μL, *p* < 0.05; *p* < 0.001 and *p* < 0.01, respectively) compared to the IL‐1β group. Likewise, cell concentrations in the 50 μg/mL group were significantly higher than the IL‐1β group at both 10 and 14 days (238.0 ± 31.4 cells/μL and 262.0 ± 36.2 cells/μL, *p* < 0.01 and *p* < 0.05, respectively).

Furthermore, WJ‐MSC‐EVs improved hNPC viability (Figure 3B). After 1 day of treatment, the LIVE/DEAD assay showed significantly fewer dead cells in the 100 μg/mL WJ‐MSC‐EV group (20.8% ± 1.2%, *p* < 0.05) compared to the IL‐1β group, which exhibited a higher percentage of dead cells (30.1% ± 3.0%, *p* < 0.05 compared to control group). These findings were further confirmed by Live/Dead staining of hNPCs encapsulated in alginate beads (Figure 3C).

### 3.3 WJ‐MSC‐EVs Attenuated Nitrite‐Induced Oxidative Stress

**FIGURE 4:**
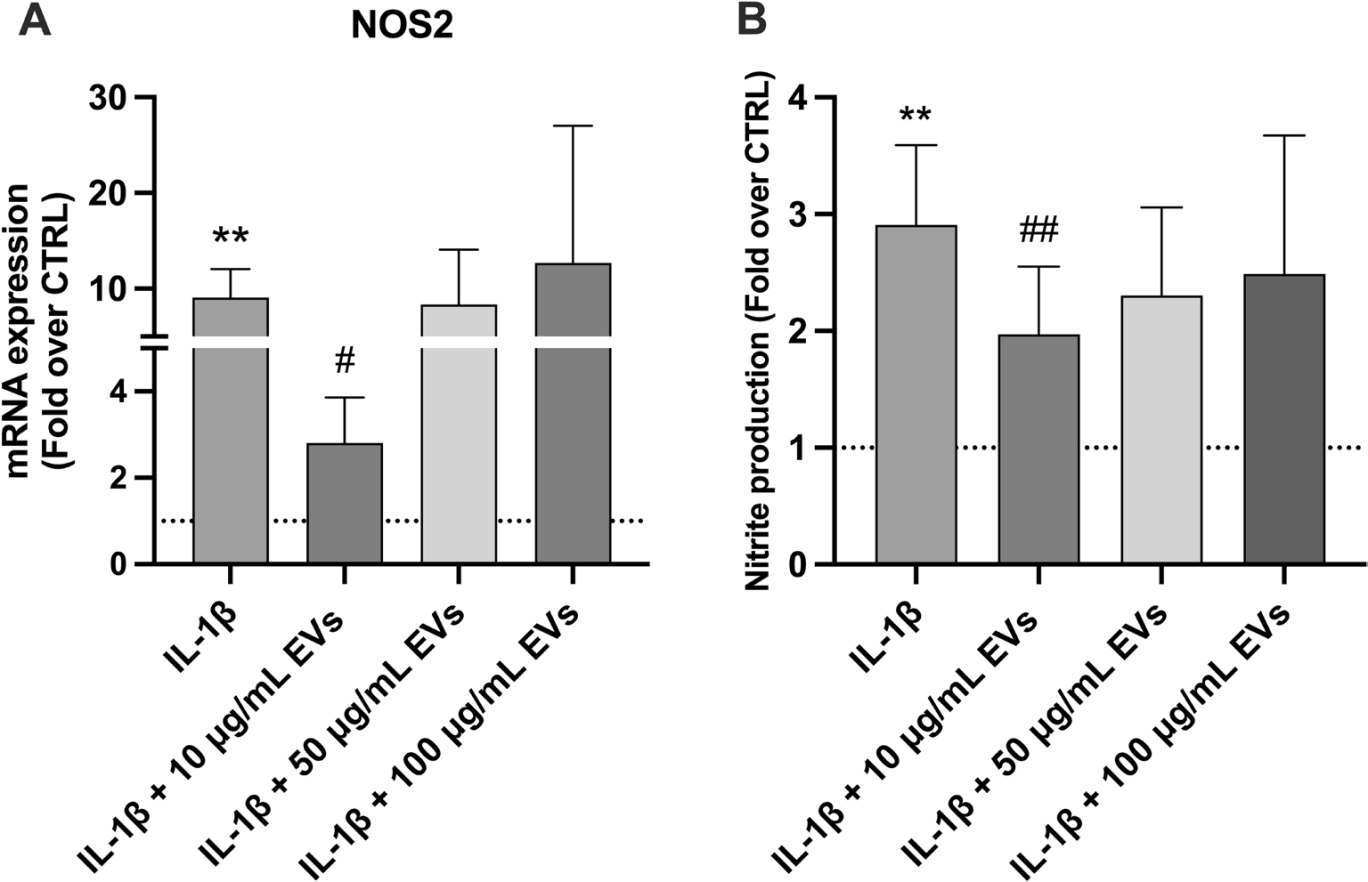
(A) NOS2 mRNA levels were significantly reduced by 10 μg/mL WJ‐MSC‐EVs (*n* = 5). EVs led to a significant decrease in nitrite release in cell supernatant (B) after 7 days in groups treated with 10 μg/mL EVs compared to the IL‐1β group (*n* = 6). Data were analyzed through one‐way ANOVA with Dunnett's multiple comparisons test, where each group was compared with IL‐1β. **p < 0.01 compared to the control group, ^#^ *p* < 0.05, ^##^ *p* < 0.01 compared to the IL‐1β group. Abbreviations: HNPCs, human nucleus pulposus cells; NOS2, nitric oxide synthase 2; WJ‐MSC‐EVs, Wharton's Jelly mesenchymal stromal cell‐derived extracellular vesicles.

After 7 days of treatment, the 10 μg/mL WJ‐MSC‐EV group showed significantly lower NOS‐2 mRNA levels (2.8 ± 1.1) compared to the IL‐1β group (*p* < 0.05; Figure 4A). Additionally, nitrite release into the supernatant, measured via the Griess reaction as an indicator of NOS‐2 gene expression, reflected these findings (Figure 4B). The fold‐change in nitrite concentration for the 10 μg/mL WJ‐MSC‐EV group was 2.0 ± 0.6. Notably, both NOS‐2 expression (9.1 ± 3.0) and nitrite levels (2.9 ± 0.7) were significantly elevated following IL‐1β exposure alone (*p* < 0.01).

### 3.4 WJ‐MSC‐EVs Enhanced ECM Production

**FIGURE 5:**
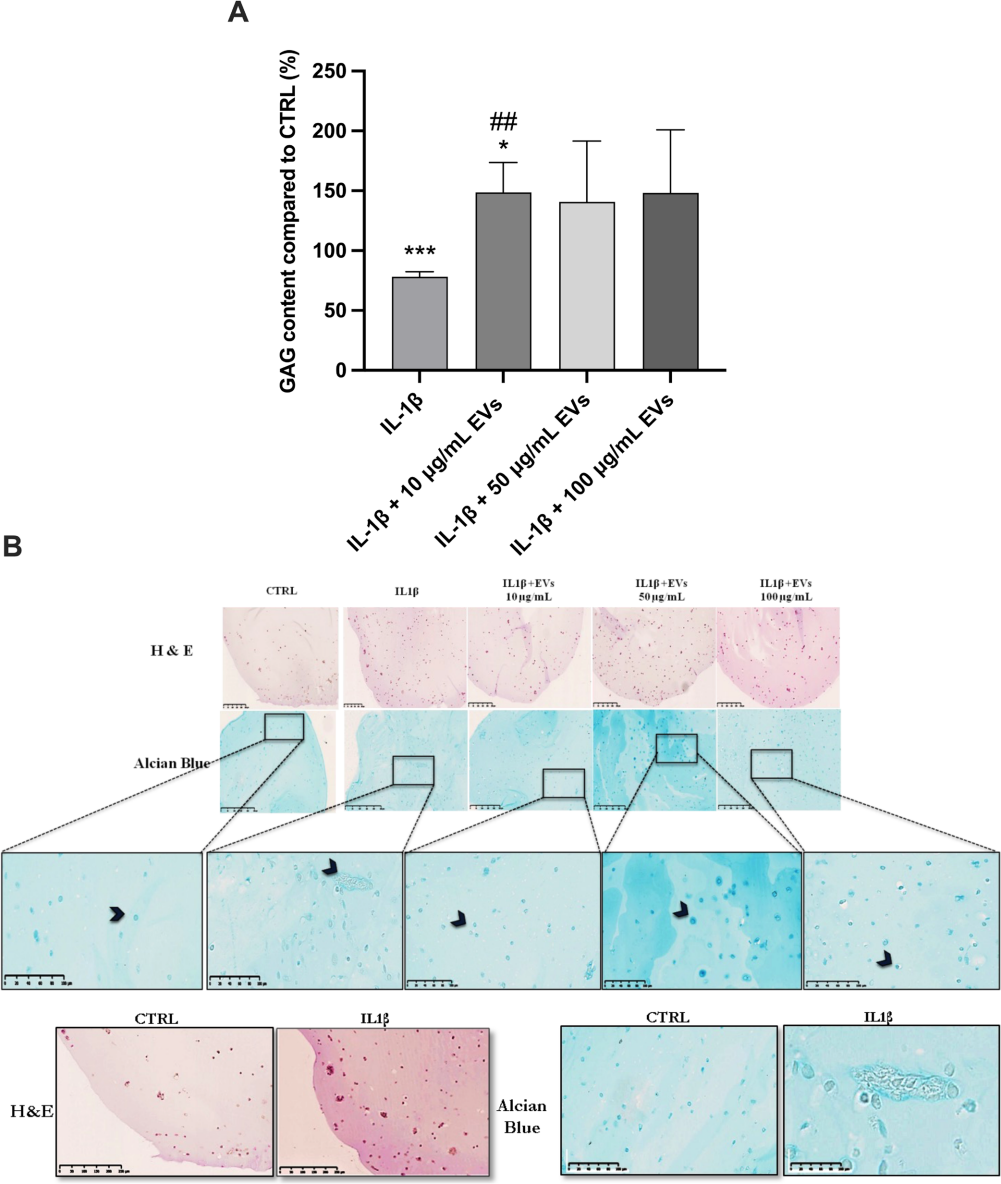
WJ‐MSC‐EVs enhanced ECM synthesis in IL‐1β‐treated hNPCs. (A) GAG/DNA ratio in hNPCs after WJ‐MSC‐EVs treatment demonstrated an increase in all experimental groups, although significant only in the 10 μg/mL WJ‐MSC‐EV group. Data were analyzed through one‐way ANOVA with Dunnett's multiple comparisons test, where each group was compared with IL‐1β. (B) Representative images of Alcian Blue staining of hNPC alginate beads are shown. Blue color indicates proteoglycans. Arrowheads point to proteoglycan deposits. Upper rows, scale bar: 500 μm; bottom boxes, scale bar: 100 μm. Data are expressed as GAG/DNA ratio percent variation between the control and experimental groups (*n* = 5). *p < 0.05, ***p < 0.001 compared to the control group, ##*p* < 0.01 compared to the IL‐1β group. Abbreviations: GAG, glycosaminoglycans; hNPCs, human nucleus pulposus cells; WJ‐MSC‐EVs, Wharton's Jelly mesenchymal stromal cell‐derived extracellular vesicles.

The DMMB colorimetric assay revealed a significant increase in GAG production normalized to DNA content in hNPCs cultured in 3D and treated with 10 μg/mL WJ‐MSC‐EVs. These cells counteracted the negative effects of IL‐1β and displayed significantly higher GAG levels compared to both control and IL‐1β groups (148.7% ± 24.9%, *p* < 0.05 and *p* < 0.01, respectively; Figure 5A). Alcian Blue staining qualitatively confirmed these results, indicating that WJ‐MSC‐EVs may stimulate ECM synthesis in degenerated and inflamed hNPCs (Figure 5B).

### 3.5 WJ‐MSC‐EVs Maintained hNPCs Phenotype and Blunted Inflammatory and ECM Catabolic Marker Expression

**FIGURE 6:**
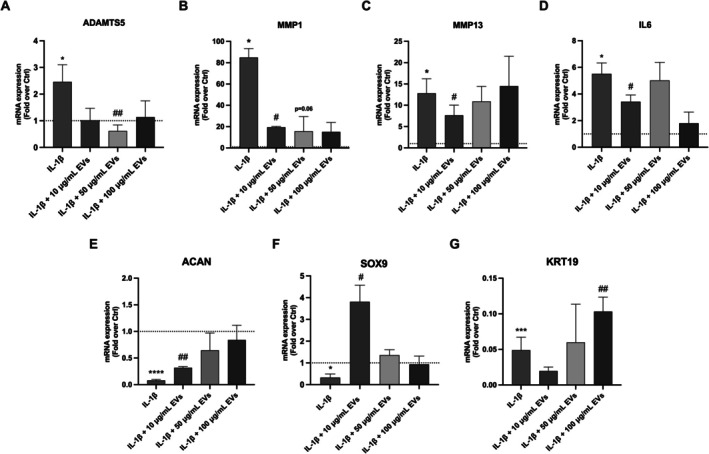
WJ‐MSC‐EVs maintained the hNPC phenotype and reduced catabolic gene expression levels. WJ‐MSC‐EV treatment resulted in a significant decrease of ADAMTS5 (A), MMP1 (B), MMP13 (C) and IL6 (D) mRNA levels, while increasing ACAN (E), SOX9 (F) and KRT19 (G) gene expression. Results were normalized based on GAPDH expression and calculated as fold change compared to the controls. Data were analyzed through one‐way ANOVA with Dunnett's multiple comparisons test, where each group was compared with IL‐1β. *p < 0.05, ***p < 0.001, ****p < 0.0001 compared to the control group, ^#^ *p* < 0.05, ^##^ *p* < 0.01 compared to the IL1‐β group. Abbreviations: ACAN, aggrecan; ADAMTS, a disintegrin and metalloproteinase with thrombospondin motifs; hNPCs, human nucleus pulposus cells; IL, interleukin; MMP, matrix metalloproteinase; NOS, nitric oxide synthase; SOX, SRY‐box transcription factor; WJ‐MSC‐EVs = Wharton's Jelly mesenchymal stromal cell‐derived extracellular vesicles.

IL‐1β stimulation significantly increased the expression of *ADAMTS‐5*, *MMP‐1*, and *MMP‐13* genes (2.5 ± 0.6; 73.0 ± 24.9; 12.8 ± 8.3, *p* < 0.05). However, *ADAMTS5* mRNA levels (Figure 6A) significantly decreased following treatment with 50 μg/mL WJ‐MSC‐EVs (0.6 ± 0.2, *p* < 0.01), whereas *MMP1* gene expression (Figure 6B) was downregulated by 10 μg/mL WJ‐MSC‐EVs (18.9 ± 1.2, *p* < 0.05). Likewise, a statistically significant decrement of *MMP13* expression (Figure 6C) was noted after exposure to 10 μg/mL WJ‐MSC‐EVs (7.7 ± 5.8, *p* < 0.05). Moreover, WJ‐MSC‐EVs were able to significantly blunt *IL6* gene expression (Figure 6D) after exposure to 10 μg/mL WJ‐MSC‐EVs (3.4 ± 1.0, *p* < 0.05) compared to IL‐1β. Interestingly, WJ‐MSC‐EV‐treated hNPCs expressed significantly higher *ACAN*, *SOX9*, and *KRT19* mRNA levels compared to the control group. hNPCs displayed increased mRNA expression levels of *ACAN* (Figure 6E), *SOX9* (Figure 6F), and *KRT19* (Figure 6G) after treatment with 10 μg/mL WJ‐MSC‐EVs compared to IL‐1β (0.3 ± 0.05; 3.8 ± 1.7 and 0.1 ± 0.04, *p* < 0.01, *p* < 0.05, *p* < 0.01, respectively). Conversely, *ACAN*, *SOX9*, and *KRT19* expression levels were significantly lower in IL‐1β‐treated hNPCs (0.1 ± 0.04; 0.3 ± 0.4; 0.04 ± 0.03, *p* < 0.0001, *p* < 0.01, *p* < 0.001, respectively).

## 4 Discussion

In this study, we demonstrated that WJ‐MSC‐EVs effectively mitigated the in vitro catabolic effects of IL‐1β on hNPCs in a 3D culture system. Treated cells exhibited enhanced proliferation and viability, reduced oxidative stress, increased GAG content, and positively modulated gene expression of ECM and phenotypic NPC markers. These findings complement our previous study [14], which reported the anabolic effects of WJ‐MSC‐EVs under basal conditions, further suggesting the potential of this EV product for treating IDD.

Current biological approaches to treat IDD primarily involve intradiscal injection of MSCs to exert a direct local anabolic effect and address the hostile microenvironment of degenerated IVDs [16, 17]. Several studies have indicated that paracrine mechanisms allow MSCs to play a crucial role in promoting tissue repair and regeneration. The secretome, comprising secreted bioactive factors and EVs, may overcome ethical issues related to cell transplantation, precarious survival, or unwanted differentiation of cells in the host IVD tissue [18, 19, 20, 21, 22, 23, 24]. In our study, we employed WJ‐MSCs as the source of MSCs. These young stem cells derived from the umbilical cord share common features with other adult MSCs, including MSC surface markers, plastic adherence, and osteogenic, chondrogenic, and adipogenic differentiation abilities [25, 26]. The use of WJ‐MSCs in clinical settings is increasingly attractive due to their high proliferation capability, phenotype maintenance over multiple passages in vitro, hypoimmunogenicity, and immunomodulatory potential compared to MSCs from the bone marrow or adipose tissue. Moreover, safe and minimally invasive collection, low incidence of graft‐versus‐host disease, and the consequent possibility of manufacturing allogeneic products offer several benefits related to healthy donors [13, 27].

In this study, we reported that WJ‐MSC‐EVs promoted hNPCs metabolism, proliferation, GAG synthesis, and reduced the mRNA levels of catabolic markers in a 3D in vitro model. hNPCs were encapsulated in alginate beads and treated with three different WJ‐MSC‐EVs concentrations, as reported in our previous study [14]. WJ‐MSC‐EVs significantly promoted cell proliferation at the lowest (10 μg/mL) and medium (50 μg/mL) concentrations after pretreatment with 10 ng/mL IL‐1β. Although all three EV doses attenuated hNPC mortality, the lowest and highest concentrations (10 and 100 μg/mL) significantly reduced the number of dead cells compared to the IL‐1β group. Several noxious stimuli including H_2_O_2_, lipopolysaccharide, IL‐1β, tumor necrosis factor (TNF)‐ɑ, acidic pH, and high glucose, have been used to mimic IDD in vitro and in vivo, wherein MSCs‐derived EVs have shown proliferative and anti‐apoptotic effects. The mechanisms underlying these effects are often related to miRNAs transported by EVs to degenerated IVD cells. Previous studies have reported that EVs isolated from bone marrow‐derived MSCs (BM‐MSCs) promoted the proliferation of degenerated NPCs by activating the SOX4/Wnt/β‐catenin axis pathway through miR‐129–5p [28]. Likewise, Wang et al. [29] found that MSC‐EVs carrying miR‐31 upregulated the nuclear factor of activated T‐cells (NFAT5)/Wnt/β‐catenin signaling pathway, thus promoting ECM synthesis and NPC proliferation. Similarly, EV‐delivered miR‐194–5p enhanced proliferation and osteogenic differentiation in TNF‐α‐treated NPCs by downregulating tumor receptor‐associated factor 6 (TRAF6) [30]. Cheng et al. [31] reported that MSC‐derived exosomes containing miR‐21 inhibited phosphatase and tensin homolog (PTEN) and activated the phosphoinositide 3‐kinase/protein kinase B (PI3K/Akt) pathway in a rat model of IDD, preventing TNF‐α‐mediated apoptosis. Excessive apoptosis of NPCs also contributes significantly to IDD progression. Recent studies have demonstrated that exosomes derived from BM‐MSCs can counteract IL‐1β‐induced NPC apoptosis by suppressing the NLRP3 inflammasome activation. Additionally, BM‐MSC‐derived EVs have been shown to inhibit ER stress‐induced apoptosis in NPCs by regulating AKT and ERK signaling pathways [32].

During IDD, the balance between oxygen free radical generation and antioxidant defense mechanisms is disrupted, leading to the overproduction of reactive oxygen species (ROS) [33]. Our findings indicate that WJ‐MSC‐EVs effectively reduced nitrite release and NOS‐2 expression in hNPCs. However, in the IL‐1β‐treated groups, the altered inflammatory microenvironment likely mitigated the beneficial effects of EVs. Supporting this, Xu et al. [34] demonstrated that platelet‐rich plasma EVs act as ROS scavengers in IDD models by activating the Keap1‐Nrf2 signaling pathway. Similarly, Hu et al. [35] reported that MSC‐derived EVs alleviate ROS production, reduce mitochondrial damage, and prevent NPC apoptosis.

The synthesis of ECM components in response to tissue damage is an essential endogenous reparative response within the IVD. Our results confirmed that WJ‐MSC‐EVs significantly enhanced GAG production, as evidenced by the DMMB assay and Alcian Blue staining. GAGs and proteoglycans provide structural support to the ECM, ensuring IVD integrity. However, an imbalance in the expression of MMPs and their inhibitors (TIMPs) can compromise ECM homeostasis [36]. In this study, WJ‐MSC‐EVs downregulated the expression of catabolic enzymes MMP‐1, MMP‐13, ADAMTS‐5, and the proinflammatory cytokine IL‐6, all implicated in IDD pathogenesis. Previous studies have also shown that BM‐MSC‐derived EVs promoted ECM synthesis via the SOX4/Wnt/β‐catenin pathway [28]. Conversely, EVs from degenerative NPCs may accelerate ECM degradation, highlighting the potential adverse effects of degenerative EVs on IDD progression [37, 38].

As an avascular tissue, the inflammatory mechanisms in the IVD remain poorly understood. Studies have demonstrated that MSC‐derived EVs exerted anti‐inflammatory effects by inhibiting NLRP3 inflammasome activation in vitro and in vivo [34]. For example, Zhu et al. [39] identified EVs delivering miR‐142‐3p as key factors in mitigating IL‐1β‐induced inflammation through MAPK signaling by targeting mixed lineage kinase 3 (MLK3). In our study, gene expression analysis revealed that WJ‐MSC‐EVs enhanced the expression of NP markers ACAN, KRT19, and SOX9 under inflammatory conditions, confirming that our 3D culture model preserved the phenotypic integrity of healthy NPCs.

The field of EV research is rapidly evolving, with significant potential for their application as nano‐drug delivery systems and biomarkers for disease screening [40]. MSC‐derived EVs offer a promising alternative to traditional cell therapy due to their biocompatibility and intrinsic therapeutic cargo, particularly miRNAs. However, challenges remain, including the influence of the hostile IVD microenvironment on EV delivery and efficacy, as well as the need for optimized isolation and purification methods in compliance with good manufacturing practices for clinical translation [41].

This study has several limitations. First, the findings were derived from in vitro experiments, which do not fully capture the complexity of IDD in vivo. Second, patient selection was not performed; hNPCs were obtained from lumbar herniated disc tissues without considering the severity of IDD, which could influence their biological response to EVs, as recently reported [42, 43]. Third, the inflammatory stimulation used in this study employed 10 ng/mL of IL‐1β. Although this concentration is widely reported in the literature, it may not accurately reflect physiological conditions in the intervertebral disc. Another key limitation of this study is the use of normoxic (21% O_2_) culture conditions, which do not accurately reflect the hypoxic environment of the intervertebral disc, where oxygen tensions are significantly lower. This discrepancy may influence cellular behavior and phenotype. Most importantly, the cargo of isolated WJ‐MSC‐EVs was not characterized, thus hampering any possible mechanistic speculations on observed effects. Further studies are warranted to address these limitations and better understand the therapeutic potential of EVs in IDD.

## 5 Conclusion

In this study, we evaluated the therapeutic effects of WJ‐MSCs EVs in hNPCs using a 3D culture model under in vitro inflammation. WJ‐MSC EVs increased cell proliferation and ECM production while reducing cell death, oxidative stress, and catabolic marker expression. Our results suggest that WJ‐MSC EVs may represent a promising cell‐free strategy for IVD regeneration.

## Author Contributions

The study was conceptualized by V.T., G.D.G, C.C., and G.V. L.A. V.T., G.D.G, C.C., and A.D. performed the experiments. The first draft of the manuscript was written by V.T., G.D.G., and C.C. L.A. F.R., A.D., R.P., G.V., and V.D. revised the advanced version of the paper. R.P. and V.D. supervised the study. All authors read and approved the final manuscript.
